# Nanoparticles in the clinic: An update post COVID‐19 vaccines

**DOI:** 10.1002/btm2.10246

**Published:** 2021-08-13

**Authors:** Aaron C. Anselmo, Samir Mitragotri

**Affiliations:** ^1^ Division of Pharmacoengineering and Molecular Pharmaceutics Eshelman School of Pharmacy, University of North Carolina at Chapel Hill Chapel Hill North Carolina USA; ^2^ John A. Paulson School of Engineering & Applied Sciences Harvard University Cambridge Massachusetts USA; ^3^ Wyss Institute for Biologically Inspired Engineering Boston Massachusetts USA

**Keywords:** clinic, clinical translation, clinical trials, drug delivery, nanomedicine, nanoparticles, translational medicine

## Abstract

Nanoparticles are used in the clinic to treat cancer, resolve mineral deficiencies, image tissues, and facilitate vaccination. As a modular technology, nanoparticles combine diagnostic agents or therapeutics (e.g., elements, small molecules, biologics), synthetic materials (e.g., polymers), and biological molecules (e.g., antibodies, peptides, lipids). Leveraging these parameters, nanoparticles can be designed and tuned to navigate biological microenvironments, negotiate biological barriers, and deliver therapeutics or diagnostic agents to specific cells and tissues in the body. Recently, with the Emergency Use Authorization of the COVID‐19 lipid nanoparticle vaccines, the advantages and potential of nanoparticles as a delivery vehicle have been displayed at the forefront of biotechnology. Here, we provide a 5‐year status update on our original “Nanoparticles in the Clinic” review (also a 2‐year update on our second “Nanoparticles in the Clinic” review) by discussing recent nanoparticle delivery system approvals, highlighting new clinical trials, and providing an update on the previously highlighted clinical trials.

## 1 INTRODUCTION

Over the past 2 years, the nanomedicine landscape has evolved rapidly, driven by the worldwide clinical introduction of the Moderna and Pfizer‐BioNTech COVID‐19 lipid nanoparticle mRNA vaccines.^1^ Given this sudden expansion of nanoparticle use in the clinic, we are updating our “Nanoparticles in the Clinic” review and providing an update on the clinical landscape of nanomedicines. Our original review was published in 2016 and highlighted >25 approved nanomedicines and >45 unapproved nanoparticles that were being evaluated clinical trials.^2^ In 2019, our second review included three new nanoparticle approvals, added >75 new clinicals trials for the previously highlighted unapproved nanoparticles, and added >15 new nanoparticles that entered clinical trials.^3^ In this 2021 update, we provide a broad overview of the current clinical landscape by adding two nanoparticles that recently received Emergency Use Authorization (both in 2020), >30 new trials that have started for previously tabulated unapproved nanoparticles, and >35 new nanoparticle technologies (associated with >55 new trials) that have recently entered clinical trials.

## 2 UPDATES ON CURRENTLY APPROVED NANOPARTICLES AND NEW ADDITIONS

Over the past 2 years, the nanomedicine landscape has evolved rapidly, driven by the global need for new technologies to provide prophylactic and therapeutic approaches against the coronavirus disease 2019 (COVID‐19),^4^, ^5^, ^6^ which is caused by the severe acute respiratory syndrome coronavirus 2 (SARS‐CoV‐2).^7^ Of the technologies that have emerged to combat COVID‐19, lipid nanoparticles are the delivery vehicle used in the Moderna and Pfizer‐BioNTech COVID‐19 vaccines, both of which were granted Emergency Use Authorization in the United States in 2020.^8^

The Moderna vaccine, mRNA‐1273, is a lipid nanoparticle consisting of the ionizable cationic lipid SM‐102 (heptadecan‐9‐yl 8 ((2 hydroxyethyl) (6 oxo 6‐(undecyloxy) hexyl) amino) octanoate), DSPC (1,2‐distearoyl‐snglycero‐3 phosphocholine), cholesterol, and PEG‐DMG (1 monomethoxypolyethyleneglycol‐2,3‐dimyristylglycerol with polyethylene glycol).^9^ mRNA‐1273 was granted Emergency Use Authorization by the U.S. FDA on December 18th, 2020,^10^ based on a number of clinical trials, including one that demonstrated anti‐SARS‐CoV‐2 immune responses in participants without trial‐limiting safety concerns^11^ and another clinical trial with 30,420 participants that demonstrated 94.1% efficacy at preventing COVID‐19 illness.^12^ The Pfizer‐BioNTech vaccine, BNT162b2, is also a lipid nanoparticle and consists of the ionizable cationic lipid ALC‐0315 ((4‐hydroxybutyl)azanediyl)bis(hexane‐6,1‐diyl)bis(2‐hexyldecanoate), DSPC (1,2‐distearoyl‐sn‐glycero‐3‐phosphocholine), cholesterol, and PEG‐DMA (2 [(polyethylene glycol)‐2000]‐N,N‐ditetradecylacetamide).^9^ BNT162b2 was granted Emergency Use Authorization by the U.S. FDA on December 11th, 2020,^10^ based on a number of clinical trials, including one that demonstrated both safety and immunogenicity of BNT162b2,^13^ and another clinical trial with 43,548 participants that demonstrated BNT162b2 was 95% effective in preventing COVID‐19.^14^ Both lipid nanoparticle formulations are administered intramuscularly in two separate doses and are used to encapsulate mRNA that encodes for the SARS‐CoV‐2 spike glycoprotein, which mediates attachment to host cells and thus enables viral entry.^11^ By encoding for this spike glycoprotein, the host generates an immune response to the presented antigenic proteins; thus, a neutralizing antibody response against SARS‐CoV‐2 is generated.^15^ In both formulations, lipid nanoparticles enable delivery of the sensitive mRNA cargo into the cytoplasm,^16^ which has been the major obstacle in translation of mRNA technologies.^17^ By overcoming the challenges of intracellular delivery using lipid nanoparticles, antigen presentation could occur and the neutralizing antibody response against SARS‐CoV‐2 was achieved. Collectively, mRNA‐1273 and BNT162b2 are used in >35 countries^10^ with an estimated 3 billion (2 billion for BNT162b2 and 1 billion for mRNA‐1273) doses to be manufactured throughout 2021.^18^

**FIGURE 1:**
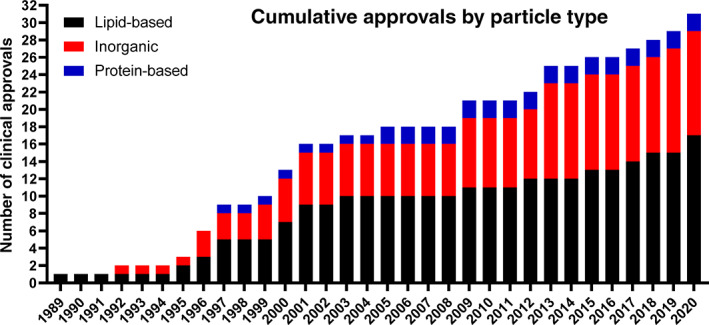
Chronological nanoparticle approvals based on particle type

**FIGURE 2:**
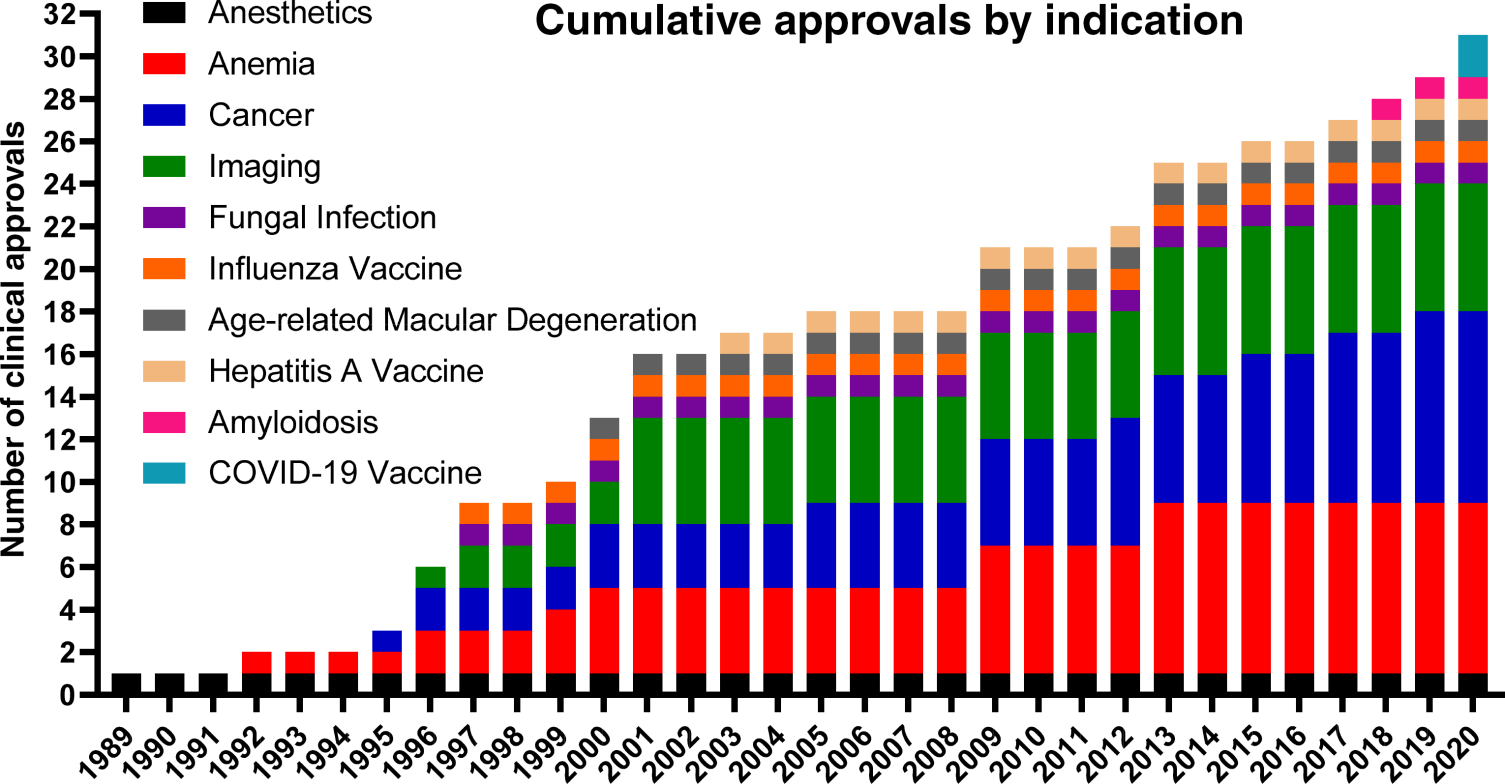
Chronological nanoparticle approvals based on indication

Table 1 has been updated to list FDA/EMA approved injectable nanomedicines up to 2021, including the lipid nanoparticle mRNA vaccines against COVID‐19. Figures 1 and 2 highlight key aspects of these tabulated findings. Figure 1 shows the chronological approvals of nanoparticles based on particle type. Lipid‐based and inorganic nanoparticles comprise the majority of clinically‐approved nanoparticles. Interestingly, the first (1989) and most recent (2020) clinically approved/authorized particles highlight how lipid‐based nanoparticles, as a platform technology, enable controlled interactions between encapsulated therapeutics and complex microenvironments within patients. While the majority of lipid‐based nanoparticles are approved and clinically‐used for intravenous applications, lipid‐based nanoparticles are also used to protect sensitive cargos (e.g., mRNA) after manufacturing, during storage, and during intramuscular muscular injection and throughout their action within the host. Figure 2 shows the chronological approvals of nanoparticles based on indication, with the dominant applications being cancer, anemia, and imaging.

**TABLE 1:** Clinically approved nanoparticle therapies and diagnostics, grouped by their broad indication

Name	Particle type	Payload	Approved application/indication	Approval (year)
*New additions*				
mRNA‐1273 (Moderna)	Lipid nanoparticle	mRNA	COVID‐19 vaccine	FDA, Emergency Use Authorization (2020)
Tozinameran/BNT162b2 (Pfizer‐BioNTech)	Lipid nanoparticle	mRNA	COVID‐19 vaccine	FDA, Emergency Use Authorization (2020)
*Cancer*				
Doxil Caelyx (Janssen)	PEGylated liposome	Doxorubicin	Ovarian cancer, HIV‐associated Kaposi's sarcoma, Multiple myeloma	FDA (1995) EMA (1996)
DaunoXome (Galen)	Liposome (non‐PEGylated)	Daunorubicin	HIV‐associated Kaposi's sarcoma	FDA (1996)
Myocet (Teva UK)	Liposome (non‐PEGylated)	Doxorubicin	Breast cancer	EMA (2000)
Abraxane (Celgene)	Albumin‐particle	Paclitaxel	Advanced non‐small cell lung cancer, Metastatic pancreatic cancer, Metastatic breast cancer	FDA (2005) EMA (2008)
Marqibo (Spectrum)	Liposome (non‐PEGylated)	Vincristine	Philadelphia chromosome‐negative acute lymphoblastic leukemia	FDA (2012)
MEPACT (Millennium)	Liposome (non‐PEGylated)	Mifamurtide	Osteosarcoma	EMA (2009)
NBTXR3 Hensify (Nanobiotix)	Hafnium oxide nanoparticles	Stimulated with external radiation to enhance tumor cell death via electron production	Squamous cell carcinoma	CE Mark (2019)
Onivyde MM‐398 (Merrimack)	PEGylated liposome	Irinotecan	Metastatic pancreatic cancer	FDA (2015)
VYXEOS CPX‐351 (Jazz Pharmaceuticals)	Liposome	Cytarabine:daunorubicin (5:1 molar ratio)	Acute myeloid leukemia	FDA (2017) EMA (2018)
*Iron‐replacement*				
CosmoFer INFeD Ferrisat (Pharmacosmos)	Iron dextran colloid	Iron	Iron deficient anemia	FDA (1992) Some of Europe
DexFerrum DexIron (American Regent)	Iron dextran colloid	Iron	Iron deficient anemia	FDA (1996)
Ferrlecit (Sanofi)	Iron gluconate colloid	Iron	Iron replacement for anemia treatment in patients with chronic kidney disease	FDA (1999)
Venofer (American Regent)	Iron sucrose colloid	Iron	Iron replacement for anemia treatment in patients with chronic kidney disease	FDA (2000)
Feraheme (AMAG) Rienso (Takeda) Ferumoxytol	Iron polyglucose sorbitol carboxymethylether colloid	Iron	Iron deficiency in patients with chronic kidney disease	FDA (2009)
Injectafer Ferinject (Vifor)	Iron carboxymaltose colloid	Iron	Iron deficient anemia	FDA (2013)
Monofer (Pharmacosmos)	10% iron isomaltoside 1000 colloid	Iron	Treating iron deficiency and anemia when oral methods do not work or when iron delivery is required immediately	Some of Europe (2009)
Diafer (Pharmacosmos)	5% iron isomaltoside 1000 colloid	Iron	Iron deficient anemia	Some of Europe (2013)
*Imaging agents*				
Definity (Lantheus Medical Imaging)	Lipid microspheres	Perflutren	Ultrasound contrast agent	FDA (2001)
Feridex I.V. (AMAG) Endorem	Iron dextran colloid	Iron	Imaging of liver lesions	FDA (1996) Discontinued (2008)
Ferumoxtran‐10 Combidex Sinerem (AMAG)	Iron dextran colloid	Iron	Imaging lymph node metastases	Only available in the Netherlands (2013)
Optison (GE Healthcare)	Human serum albumin stabilized microspheres	Perflutren	Ultrasound contrast agent	FDA (1997) EMA (1998)
SonoVue (Bracco Imaging)	Phospholipid stabilized microbubble	Hexafluoride	Ultrasound contrast agent	EMA (2001)
Resovist (Bayer Schering Pharma) Cliavist	Iron carboxydextran colloid	Iron	Imaging of liver lesions	Some of Europe (2001) Discontinued (2009)
*Vaccines*				
Epaxal (Crucell)	Liposome	Inactivated hepatitis A virus	Hepatitis A vaccine	Some of Europe (2003; Now discontinued)
Inflexal V (Crucell)	Liposome	Trivalent‐influenza virus surface antigens	Influenza vaccine	Some of Europe (1997; Now discontinued)
*Anesthetics*				
Diprivan	Liposome	Propofol	Induction and maintenance of sedation or anesthesia	FDA (1989)
*Amyloidosis*				
ONPATTRO Patisiran ALN‐TTR02 (Alnylam Pharmaceuticals)	Lipid nanoparticle	RNAi for the knockdown of disease‐causing TTR protein	Transthyretin (TTR)‐mediated amyloidosis	FDA (2018) EMA (2018)
*Fungal infections*				
AmBisome (Gilead Sciences)	Liposome	Amphotericin B	Cryptococcal Meningitis in HIV‐infected patients Aspergillus, Candida and/or Cryptococcus species infections (secondary) Visceral leishmaniasis parasite in immunocompromised patients	FDA (1997) Most of Europe
*Macular degradation*				
Visudyne (Bausch and Lomb)	Liposomal	Verteporfin	Treatment of subfoveal choroidal neovascularization from age‐related macular degeneration, pathologic, or ocular histoplasmosis	FDA (2000) EMA (2000)

*Note*: Recent nanoparticles that have received Emergency Use Authorization are separately listed in the first rows.

**FIGURE 3:**
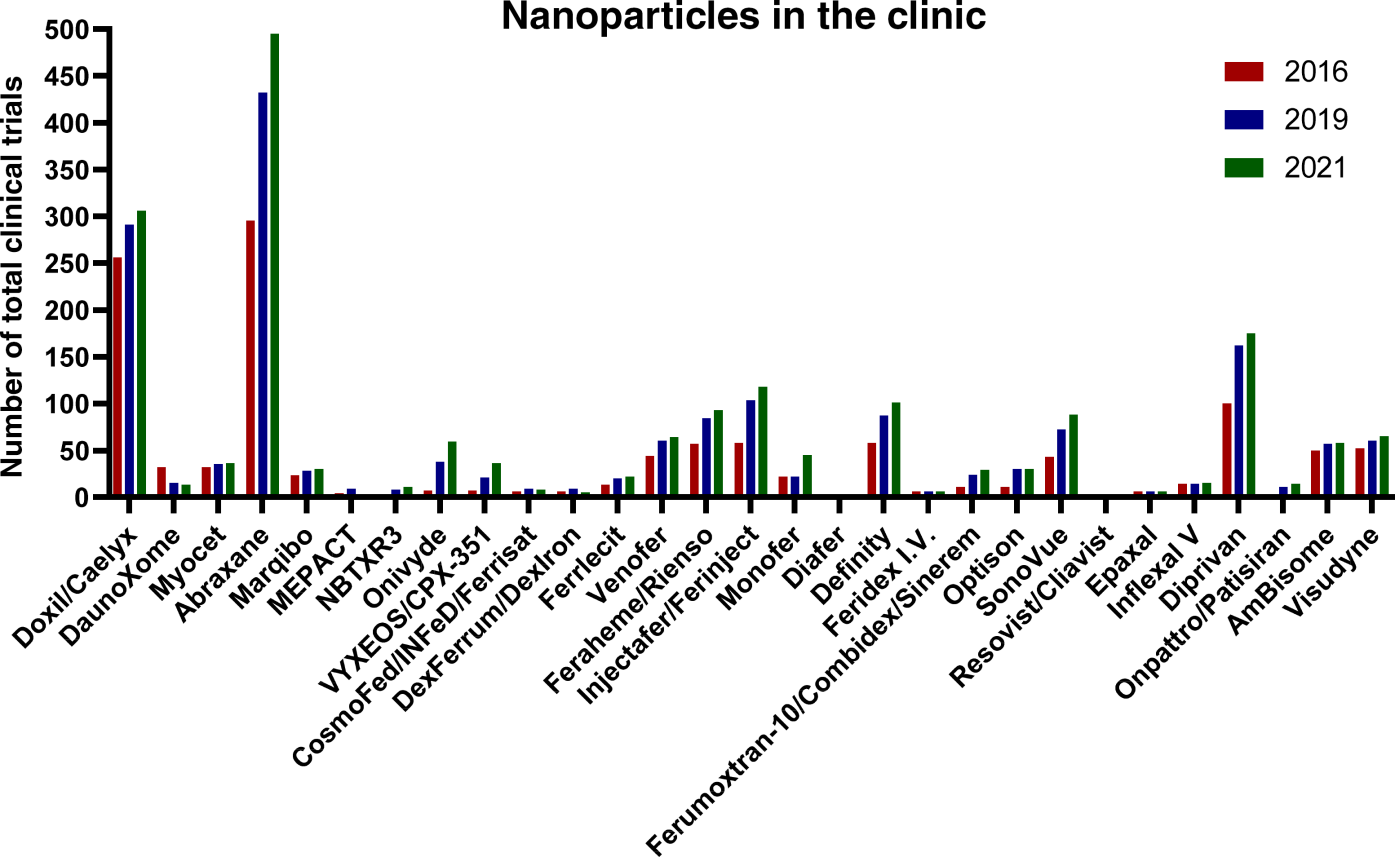
Chronological update on the number of clinical trials for each nanoparticle, based on the number of identified trials in our previous “Nanoparticles in the Clinic” reviews in 2016^2^ and 2019.^3^ These trials and nanoparticles have appeared on the ClinicalTrial.gov database *Source*: Modified with permissions from References 2 and 3

In addition to this updated Table 1, we also report an update on the number of clinical trials for approved nanoparticles that have appeared since our previous two articles in 2016 and 2019^2^, ^3^: Figure 3 shows the number of clinical trials for each of the approved nanoparticles (see Table S1 for detailed summary) from 2016 (red) to 2019 (blue) to 2021 (green). Of particular note, we observe: (i) an increase in the number of clinical trials for 21 of the 29 approved nanoparticles in Table 1 (excluding the newly‐added mRNA‐1723 and BNT162b2), and (ii) an increase in the total number of clinical trials for approved nanoparticles from 1220 (in 2016), to 1716 (in 2019), and to 1935 (in 2021). Together, this demonstrates the continued success of nanoparticles that are being introduced into the clinic and the continued investigation of already‐approved nanoparticles toward expanding or improving their clinical use.

## 3 UPDATE ON PREVIOUS CLINICAL TRIALS FOR UNAPPROVED NANOPARTICLES

In our previous article,^3^ >60 different non‐approved nanoparticles were listed as active in >100 clinical trials. Here, we are updating the current clinical landscape for each of these clinically investigated nanoparticles (Table 2). In this update, >40 new trials have been added. Regarding previous clinical trials that were available before 2019,^3^ 11 have been updated to active status, 5 have been updated to recruiting status, 5 have been terminated, 8 have been updated to unknown status, 12 have been completed, 3 have been withdrawn, and 12 have posted results that are viewable on ClinicalTrials.gov (Table 2). Of particular note: (i) 10 new trials began for a mitoxantrone hydrochloride liposome, used for the treatment of various cancers, (ii) 11 new trials began for Sonazoid, which is a lipid‐encapsulated formulation of F‐butane for ultrasound imaging, and (iii) results have been posted for PNT2258, DCR‐MYC, SGT‐53, NK105, CRLX101, CRLX301, AuroLase, and Halaven. Table 2 provides a detailed tabulation of these recent updates.

**TABLE 2:** Updates on previously reported nanoparticle clinical trials that are not clinically approved or authorized and are currently active in clinical trials

Name (company)	Particle type	Payload	Investigated application/indication	ClinicalTrials.gov identifiers (phase)	Updates since 2019
*Lipid‐based (cancer)*					
PROMITIL (Lipomedix Pharmaceuticals)	PEGylated liposome	Mitomycin‐C	Solid tumors	2016: NCT01705002 (Ph I): Completed 2019: NCT03823989 (Ph Ib): Recruiting (Active as of 2021) 2021 additions: NCT04729205 (Ph I): Recruiting	2021: One new trial One trial updated to active 2019: One new trial One trial updated to completed
ThermoDox® (Celsion)	Lyso‐thermosensitive liposome	Doxorubicin	Various cancers	2016: NCT02536183 (Ph I): Recruiting NCT00826085 (Ph I/II): Completed NCT02112656 (Ph III): Completed NCT02181075 (Ph I): Completed 2019 additions: NCT03749850 (Ph I): Not yet recruiting (Recruiting as of 2021) 2021 additions: NCT04852367 (Ph I): Not yet recruiting NCT04791228 (Ph II): Not yet recruiting	2021: Two new trials One trial updated to recruiting 2019: One new trial Three trials updated to completed
Oncoprex/GPX‐001 (Genprex)	Liposome	FUS1 (TUSC2)	Lung cancer	2016: NCT01455389 (Ph I/II): Active, not recruiting 2021 additions: NCT04486833 (Ph I/II): Not yet recruiting	2021: One new trial 2019: No updates
Halaven E7389‐LF (Eisai)	Liposome	Eribulin mesylate	Solid tumors	2016: NCT01945710 (Ph I): Completed 2019 additions: NCT03207672 (Ph I): Recruiting (Active as of 2021) 2021 additions: NCT04078295 (Ph I/II): Recruiting	2021: One new trial One trial updated to recruiting Results posted and viewable on ClinicalTrials.gov for NCT01945710 2019: One new trial One trial updated to completed
^188^Re‐BMEDA‐liposome	Pegylated liposome	188Re‐N,N‐bis (2‐mercaptoethyl)‐N′,N′‐diethylethylenediamine	Advanced solid tumors	2016: NCT02271516 (Ph I): Unknown (Terminated as of 2021 due to concerns of accumulation of radioactivity in both the liver and spleen)	2021: One trial updated to terminated 2019: Zero new trials
Mitoxantrone Hydrochloride Liposome (CSPC ZhongQi Pharmaceutical Technology)	Liposome	Mitoxantrone	Various cancers	2016: NCT02131688 (Ph I): Unknown NCT02596373 (Ph II): Recruiting (Unknown status as of 2021) NCT02597387 (Ph II): Recruiting (Unknown status as of 2021) NCT02595242 (Ph I): Withdrawn NCT02597153 (Ph II): Terminated (Only one subject enrolled in 1.5 years) 2019 additions: NCT03776279 (Ph I): Recruiting (Unknown status as of 2021) 2021 additions: NCT04668690 (Ph III): Not yet recruiting NCT04718402 (Ph I): Recruiting NCT04902027 (Ph I): Not yet recruiting NCT04719065 (Ph I): Recruiting NCT04718376 (Ph I): Recruiting NCT04900766 (Ph I): Not yet recruiting NCT04548700 (Ph I): Not yet recruiting NCT04509466 (Ph I): Not yet recruiting NCT04331743 (Ph I): Not yet recruiting NCT04352413 (Ph II): Recruiting	2021: 10 new trials Three trials updated to unknown status 2019: One new trial One trial updated to withdrawn One trial updated to terminated
JVRS‐100	Cationic liposome	Plasmid DNA complex for immune system stimulation	Leukemia	2016: NCT00860522 (Ph I): Completed	2021: No updates 2019: Zero new trials One trial updated to completed
Lipocurc (SignPath Pharma)	Liposome	Curcumin	Solid tumors	2016: NCT02138955 (Ph I/II): Unknown	2021: No updates 2019: Zero new trials One trial updated to unknown status
LiPlaCis (LiPlasome Pharma)	Liposome with specific degradation‐controlled drug release via phospholipase A2 (PLA2)	Cisplatin	Advanced or refractory tumors	2016: NCT01861496 (Ph I): Recruiting (Active as of 2021)	2021: One trial updated to active 2019: No updates
MM‐302 (Merrimack Pharmaceuticals)	HER2‐targeted PEGylated liposome	Doxorubicin	Breast cancer	2016: NCT01304797 (Ph I): Unknown NCT02213744 (Ph II/III): Terminated (Felt not to show benefit over control per DMC and confirmed via futility analysis) 2019 additions: NCT02735798 (Ph I): Withdrawn (The study was not started due to the sponsor choosing to not fund the trial)	2021: No updates 2019: One new trial (withdrawn) One trial updated to terminated One trial updated to unknown status
LIPUSU® (Nanjing Luye Sike Pharmaceutical Co., Ltd.)	Liposome	Paclitaxel	Advanced solid tumors, or gastric, breast cancer	2016: NCT01994031 (Ph IV): Unknown NCT02142790 (Ph IV): Unknown NCT02163291 (Ph II): Unknown NCT02142010 (Not Provided): Unknown 2019 additions: NCT02996214 (Ph IV): Not yet recruiting (Active as of 2021)	2021: One trial updated to active 2019: One new trial
TKM‐080301 (Arbutus Biopharma)	Lipid particle targeting polo‐like kinase 1 (PLK1)	siRNA	Hepatocellular carcinoma	2016: NCT02191878 (Ph I/II): Completed	2021: No updates 2019: Zero new trials One trial updated to completed
siRNA‐EphA2‐DOPC	Liposome	siRNA for EphA2 knockdown	Solid tumors	2016: NCT01591356 (Ph I): Recruiting (Active as of 2021)	2021: One trial updated to active 2019: Zero new trials
PNT2258 (ProNAi Therapeutics)	Lipid nanoparticle	Proprietary single‐stranded DNAi (PNT100)	Lymphomas	2016: NCT02378038 (Ph II): Terminated NCT02226965 (Ph II): Unknown (Completed as of 2021) NCT01733238 (Ph II): Completed	2021: Results posted and viewable on ClinicalTrials.gov for NCT02378038, NCT02226965, and NCT01733238 2019: Zero new trials One trial updated to completed One trial updated to terminated One trial updated to unknown status
BP1001/Prexigebersen (Bio‐Path Holdings)	Neutral liposomes	Growth factor receptor bound protein‐2 (Grb‐2) antisense oligonucleotide	Leukemias and solid tumors	2016: NCT01159028 (Ph I): Active, not recruiting (Updated to completed) 2019 additions: NCT02923986 (Ph I): Recruiting (Withdrawn as of 2021 due to no enrollment) NCT02781883 (Ph II): Recruiting 2021 additions: NCT04196257 (Ph I): Not yet recruiting	2021: One new trial One trial updated to completed One trial updated to withdrawn 2019: Two new trials
DCR‐MYC (Dicerna Pharmaceuticals)	Lipid nanoparticle	DsiRNA for NYC oncogene silencing	Solid tumors, multiple myeloma, lymphoma, or hepatocellular carcinoma	2016: NCT02110563 (Ph I): Terminated (Sponsor Decision) NCT02314052 (Ph I/II) Terminated (Sponsor Decision)	2021: Results posted and viewable on ClinicalTrials.gov for NCT02314052 2019: Zero new trials Two trials updated to terminated
Atu027 (Silence Therapeutics GmbH)	Liposome	AtuRNAi for PKN3 knockdown in vascular endothelium	Pancreatic cancer	2016: NCT01808638 (Ph I/II): Completed	2021: No updates 2019: Zero new trials One trial completed
SGT‐53 (SynerGene Therapeutics)	Cationic liposome with anti‐transferrin receptor antibody	Wildtype p53 sequence	Glioblastoma, solid tumors, or pancreatic cancer	2016: NCT02354547 (Ph I): Recruiting (Active as of 2021) NCT02340156 (Ph II): Recruiting (Terminated as of 2021) NCT00470613 (Ph I): Completed 2019 additions: NCT03554707 (Ph I): Not yet recruiting 2021 additions: NCT02340117 (Ph II): Recruiting	2021: One new trial Results posted and viewable on ClinicalTrials.gov for NCT02340156 2019: One new trial One trial updated to completed
SGT‐94 (SynerGene Therapeutics)	Liposome with anti‐transferrin receptor antibody	RB94 plasmid DNA	Solid tumors	2016: NCT01517464 (Ph I): Completed	2021: No updates 2019: Zero new trials One trial updated to completed
MRX34 (Mirna Therapeutics)	Liposome	Double‐stranded RNA mimic of miR‐34	Liver cancer	2016: NCT01829971 (Ph I): Terminated (Five immune related serious adverse events) 2019 additions: NCT02862145 (Ph I): Withdrawn (five immune related serious adverse events in Phase 1 study)	2021: No updates 2019: One new trial (withdrawn) One trial updated to terminated
TargomiRs (EnGeneIC)	Anti‐EGFR bispecific antibody minicells (bacteria derived nanoparticles)	miR‐16 based microRNA	Mesothelioma and non‐small cell lung cancer	2016: NCT02369198 (Ph I): Completed	2021: No updates 2019: Zero new trials One trial updated to completed
MM‐310 (Merrimack Pharmaceuticals)	Liposome functionalized with antibodies targeted to the EphA2 receptor	Docetaxel	Solid tumors	2019: NCT03076372 (Ph I): Recruiting (Unknown as of 2021)	2021: No updates
EGFR(V)‐EDV‐Dox (EnGeneIC)	Bacterially derived minicell	Doxorubicin	Recurrent glioblastoma	2019: NCT02766699 (Ph I): Recruiting	2021: No updates
Alprostadil liposome (CSPC ZhongQi Pharmaceutical Technology)	Liposome	Alprostadil	Safety and tolerability	2019: NCT03669562 (Ph I): Recruiting (Unknown as of 2021) 2021 additions: NCT04197323 (Ph II): Recruiting	2021: One trial updated to active One trial updated to unknown status
Liposomal Annamycin (Moleculin Biotech)	Liposome	Annamycin	Acute myeloid leukemia	2019: NCT03388749 (Ph II): Recruiting NCT03315039 (Ph II): Recruiting (Active as of 2021) 2021 additions: NCT04887298 (Ph I/II): Not yet recruiting	2021: One new trial One trial updated to active
FF‐10832 (Fujifilm Pharmaceuticals)	Liposome	Gemcitabine	Advanced solid tumors	2019: NCT03440450 (Ph I): Recruiting	2021: No updates
Anti‐EGFR‐IL‐dox (Swiss Group for Clinical Cancer Research; University Hospital, Basel, Switzerland)	Anti‐EGFR immunoliposome	Doxorubicin	Advanced triple negative EGFR positive breast cancer High grade gliomas	2019: NCT02833766 (Ph II): Recruiting (Active as of 2021) NCT03603379 (Ph I): Recruiting (Completed as of 2021)	2021: One trial updated to active One trial updated to completed
TLD‐1/Talidox (Swiss Group for Clinical Cancer Research)	Liposome	Doxorubicin	Advanced solid tumors	2019: NCT03387917 (Ph I): Recruiting	2021: No updates
NC‐6300 (NanoCarrier)	Micelle	Epirubicin	Advanced solid tumors or soft tissue sarcoma	2019: NCT03168061 (Ph II): Recruiting	2021: No updates
MRT5201 (Translate Bio)	PEGylated liposomes	mRNA	Ornithine transcarbamylase deficiency	2019: NCT03767270 (Ph I): Not yet recruiting (Withdrawn as of 2021 due to program discontinuation)	2021: One trial updated to withdrawn
Lipo‐MERIT (BioNTech SE)	Liposome	Four naked ribonucleic acid (RNA)‐drug products	Cancer vaccine for advanced melanoma	2019: NCT02410733 (Ph I): Recruiting (Active as of 2021)	2021: One trial updated to active
BNT114/IVAC_W_bre1_uID (BioNTech SE)	Patient‐specific liposome (specificity for antigen‐expression on a patient's tumor)	Complexed RNA	Triple negative breast cancer	2019: NCT02316457 (Ph I): Recruiting (Active as of 2021)	2021: One trial updated to active
*Lipid‐based (other)*					
ND‐L02‐s0201 (Nitto Denko)	Lipid nanoparticle conjugated to Vitamin A	siRNA	Hepatic fibrosis and pulmonary fibrosis	2016: NCT02227459 (Ph I): Completed 2019 additions: NCT01858935 (Ph I): Completed NCT03241264 (Ph I): Completed NCT03538301 (Ph II): Recruiting	2021: No updates 2019: Three new trials (two completed) One trial updated to completed
ARB‐001467 TKM‐HBV (Arbutus Biopharma)	Lipid particle	Three RNAi therapeutics that target three sites on the HBV genome	Hepatitis B	2016: NCT02631096 (Ph II): Completed	2021: No updates 2019: Zero new trials One trial updated to completed
CAL02 (Combioxin SA)	Cholesterol liposomes for toxin neutralization	Sphingomyelin	Pneumonia	2016: NCT02583373 (Ph I): Completed	2021: No updates 2019: Zero new trials One trial updated to completed
Nanocort/Sunpharma1505 (Enceladus in collaboration with Sun Pharma Global)	PEGylated liposome	Prednisolone	Rheumatoid arthritis and hemodialysis fistula maturation	2016: NCT02495662 (Ph II): Terminated (Slow inclusion) NCT02534896 (Ph III): Terminated	2021: No updates 2019: Zero new trials Two trials updated to terminated
RGI‐2001 (Regimmune)	Liposome	α‐GalCer	Mitigating graft versus host disease following stem cell transplant	2016: NCT01379209 (Ph I/II): Unknown (Completed as of 2021) 2019 additions: NCT04014790 (Ph II): Not yet recruiting (Recruiting as of 2021) 2021 additions: NCT04473911 (Ph I): Recruiting	2021: One new trial One trial updated to completed One trial updated to recruiting 2019: One new trial
Sonazoid	Lipid shell	F‐butane	Contrast enhanced ultrasound for imaging hepatocellular carcinoma, skeletal muscle perfusion, or for estimating portal hypertension	2016: NCT00822991 (Not Provided): Recruiting (Unknown status as of 2021) NCT02398266 (Ph II): Unknown NCT02188901 (Not Provided): Completed NCT02489045 (Ph IV): Recruiting (Completed as of 2021) 2021 additions: In 2021, there are 28 total studies	2021: 28 trials total (11 active trials were added after 2019) 2019: Zero new trials One trial updated to unknown status One trial completed
mRNA‐1944 (Moderna)	Moderna's proprietary lipid nanoparticle technology	Two mRNAs that encode heavy and light chains of anti‐Chikungunya antibody	Safety, tolerability, pharmacokinetics and pharmacodynamics toward the prevention of Chikungunya virus infection	2019: NCT03829384 (Ph I): Recruiting (Active as of 2021)	2021: One trial updated to active
*Polymeric and micelles (cancer)*					
AZD2811 (AstraZeneca with BIND Therapeutics)	BIND therapeutics polymer particle accruing platform	Aurora B kinase inhibitor	Advanced solid tumors	2016: NCT02579226 (Ph I): Recruiting (Completed as of 2021) 2019 additions: NCT03366675 (Ph II): Terminated (Early detection of the purpose of the study) NCT03217838 (Ph I): Recruiting (Completed as of 2021) 2021 additions: NCT04525391 (Ph II): Recruiting NCT04745689 (Ph II): Recruiting	2021: Two new trials Two trials updated to completed 2019: Two new trials (one terminated)
BIND‐014 (BIND Therapeutics)	PSMA targeted (via ACUPA) PEG‐PLGA or PLA–PEG particle	Docetaxel	Prostate, metastatic, non‐small cell lung, cervical, head and neck, or KRAS positive lung cancers	2016: NCT02479178 (Ph II): Terminated NCT02283320 (Ph II): Completed NCT01812746 (Ph II): Completed NCT01792479 (Ph II): Completed NCT01300533 (Ph I): Completed	2021: No updates 2019: Zero new trials Four trials updated to completed
Cynviloq IG‐001 (Sorrento)	Polymeric micelle	Paclitaxel	Breast cancer	2016: NCT02064829 (Not Provided): Completed	2021: No updates 2019: Zero new trials One trial updated to completed
Genexol‐PM (Samyang Biopharmaceuticals)	Polymeric micelle	Paclitaxel	Head and neck or breast cancer	2016: NCT01689194 (Ph II): Unknown (Completed as of 2021) NCT02263495 (Ph II): Completed NCT00912639 (Ph IV): Unknown 2019 additions: NCT02739633 (Ph II): Recruiting (Unknown as of 2021) NCT03008512 (Ph II): Recruiting (Terminated as of 2021, due to poor accrual)	2021: One trial updated to unknown status One trial updated to terminated 2019: Two new trials One trial updated to completed One trial updated to unknown status
NC‐6004 Nanoplatin (Nanocarrier)	Polyamino acid and PEG micellar nanoparticle	Cisplatin	Advanced solid tumors, lung, biliary, bladder, or pancreatic cancers	2016: NCT02240238 (Ph I/II): Active, not recruiting (Completed as of 2021) NCT02043288 (Ph III): Unknown (Completed as of 2021) 2019 additions: NCT03771820 (Ph II): Not yet recruiting (Recruiting as of 2021) NCT03109158 (Ph I): Completed NCT02817113 (Ph I): Unknown (Terminated as of 2021 due to strategy change)	2021: Two trials updated to completed One trial updated to recruiting One trial updated to terminated 2019: Three new trials One trial updated to unknown status
NC‐4016 DACH‐Platin micelle (Nanocarrier)	Polyamino acid and PEG micellar nanoparticle	Oxaliplatin	Advanced solid tumors or lymphomas	2016: NCT01999491 (Ph I): Completed	2021: No updates 2019: Zero new trials
NK105 (Nippon Kayaku)	Micelle	Paclitaxel	Breast cancer	2016: NCT01644890 (Ph III): Completed	2021: Results posted and viewable on ClinicalTrials.gov for NCT01644890 2019: Zero new trials One trial completed
Docetaxel‐PM DOPNP201 (Samyang Biopharmaceuticals)	Micelle	Docetaxel	Head and neck cancer and advanced solid tumors	2016: NCT02639858 (Ph II): Recruiting (Unknown as of 2021) NCT02274610 (Ph I): Completed 2019 additions: NCT03585673 (Ph II): Recruiting (Unknown as of 2021) 2021 additions: NCT04066335: Recruiting	2021: One new trial Two trials updated to unknown 2019: One new trial One trial updated to completed
CriPec (Cristal Therapeutics)	Micelle	Docetaxel	Solid tumors, ovarian cancer	2016: NCT02442531 (Ph I): Completed 2019 additions: NCT03712423 (Ph I): Recruiting (Completed as of 2021) NCT03742713 (Ph II): Recruiting (Completed as of 2021)	2021: Two trials updated to completed 2019: Two new trials One trial updated to completed
CRLX101 (Cerulean)	Cyclodextrin based nanoparticle	Camptothecin	Ovarian, renal cell, small cell lung, or rectal cancers	2016: NCT02187302 (Ph II): Completed NCT02010567 (Ph I/II): Active, not recruiting (Terminated as of 2021 due to funding partner's request) NCT02389985 (Ph I): Terminated (Company decision) NCT01803269 (Ph II): Terminated (Due to lack of activity and slow accrual) NCT01652079 (Ph II): Completed 2019 additions: NCT02769962 (Ph I): Recruiting NCT03531827 (Ph II): Recruiting NCT02648711(Ph I): Terminated (Company decision) NCT01380769 (Ph II): Completed NCT01612546 (Ph II): Completed NCT00333502 (Ph II): Completed NCT01625936 (Ph I): Completed NCT00753740 (Ph II): Withdrawn (Poor trial recruitment) NCT00163319 (Ph III): Completed	2021: One trial updated to terminated Results posted and viewable on ClinicalTrials.gov for NCT01380769, NCT01803269, and NCT02010567 2019: Nine new trials (one terminated, one withdrawn, five completed) Two trials updated to completed Two trials updated to terminated
CRLX301 (Cerulean)	Cyclodextrin based nanoparticle	Docetaxel	Dose escalation study in advanced solid tumors	2016: NCT02380677 (Ph I/II): Terminated (Company decision)	2021: Results posted and viewable on ClinicalTrials.gov for NCT02380677 2019: Zero new trials One trial updated to terminated
MTL‐CEBPA (Mina Alpha)	SMARTICLES (amphoteric liposomes)	Double stranded RNA	Advanced liver cancer and solid tumors	2019: NCT02716012 (Ph I): Recruiting (Active as of 2021) 2021 additions: NCT04105335 (Ph I): Recruiting NCT04710641 (Ph II): Not yet recruiting	2021: Two new trials One trial updated to active
Imx‐110 (Immix Biopharma Australia)	Micelle	Stat3/NF‐kB/poly‐tyrosine kinase inhibitor and low‐dose doxorubicin	Advanced solid tumors	2019: NCT03382340 (Ph I/II): Recruiting	2021: No updates
IT‐141 (Intezyne Technologies)	Micelle	SN‐38	Advanced cancer	2019: NCT03096340 (Ph I): Recruiting (terminated as of 2021)	2021: One trial updated to terminated
*Polymeric and micelles (other)*					
RadProtect (Original BioMedicals)	PEG, iron, and amifostine micelle Transferrin‐mediated chelation for amifostine release	Amifostine	Dose escalation and safety for acute radiation syndrome	2016: NCT02587442 (Ph I): Unknown	2021: No updates 2019: Zero new trials
*Albumin‐bound (cancer)*					
ABI‐009 (Aadi with Celgene)	Albumin‐bound drug nanoparticle	Rapamycin	Bladder cancer, PEComa, or pulmonary arterial hypertension	2016: NCT02009332 (Ph I/II): Recruiting (Completed as of 2021) NCT02587325 (Ph I): Recruiting NCT02494570 (Ph II): Active not recruiting 2019 additions: NCT03747328 (Ph II): Not yet recruiting NCT03657420 (Ph I): Not yet recruiting (Withdrawn as of 2021) NCT03670030 (Ph II): Recruiting (Completed as of 2021) NCT03646240 (Ph I): Recruiting NCT03190174 (Ph I): Recruiting NCT00635284 (Ph I): Completed NCT03817515: Expanded Access Status: Available NCT03439462 (Ph II): Recruiting NCT03463265 (Ph II): Recruiting NCT03660930 (Ph I): Recruiting NCT02975882 (Ph I): Recruiting NCT02646319 (Ph I): Completed	2021: Two trials updated to completed 2019: 12 new trials (two completed)
ABI‐011 (NantBioScience)	Albumin‐bound drug nanoparticle	Thiocolchicine analog (IDN 5405)	Solid tumors or lymphomas	2016: NCT02582827 (Ph I): Recruiting (Withdrawn as of 2021 due to enrollment not initiated)	2021: One trial updated to withdrawn 2019: Zero new trials
*Inorganic (cancer)*					
AuroLase (Nanospectra Biosciences)	PEG‐coated silica‐gold nanoshells	Thermal ablation from near infrared light stimulation	Thermal ablation of solid primary and/or metastatic lung tumors	2016: NCT01679470: Terminated 2019 additions: NCT02680535: Recruiting (Completed as of 2021) NCT00848042: Completed 2021 additions: NCT04240639: Recruiting	2021: One new trial One trial updated to completed Results posted and viewable on ClinicalTrials.gov for NCT00848042 2019: Two new trials One trial updated to terminated
Cornell Dots	Silica nanoparticles with a NIR fluorophore, PEG coating, and a ^124^I radiolabeled cRGDY targeting peptide	NIR fluorophore	Imaging of melanoma and malignant brain tumors	2016: NCT01266096: Active, not recruiting 2019 additions: NCT03465618 (Ph I): Recruiting NCT02106598 (Ph II): Recruiting 2021 additions: NCT04167969 (Ph I): Recruiting	2021: One new trial 2019: Two new trials
Magnablate	Iron nanoparticles	Iron	Thermal ablation for prostate cancer	2016: NCT02033447 (Ph I): Completed	2021: No updates 2019: Zero new trials One trial updated to completed
NU‐0129 (Northwestern)	Spherical nucleic acid platform consisting of nucleic acids arranged on the surface of a spherical gold nanoparticle	Nucleic acids	Glioblastoma	2019: NCT03020017 (Ph I): Active, not recruiting (Completed as of 2021)	2021: No updates
*Imaging*					
AGuIX (National Cancer Institute, France)	Polysiloxane gadolinium chelates based nanoparticles	Gadolinium chelates	Various cancers	2019: NCT03308604 (Ph I): Recruiting (Unknown as of 2021) 2021 additions: NCT04881032 (Ph I/II): Not yet recruiting NCT03818386 (Ph II): Recruiting NCT04899908 (Ph II): Not yet recruiting NCT04094077 (Ph II): Active, not recruiting NCT04789486 (Ph I/II): Not yet recruiting NCT04784221 (Ph II): Not yet recruiting	2021: Six new trials One trial updated to unknown status
ONM‐100 (OncoNano Medicine)	Micelle covalently conjugated to indocyanine green	Indocyanine green	Intraoperative detection of cancer	2019: NCT03735680 (Ph II): Not yet recruiting (Recruiting as of 2021)	2021: One trial updated to recruiting

*Note*: These trials are grouped by particle type and indication.

## 4 NEW NANOPARTICLE TECHNOLOGIES IN CLINICAL TRIALS

Since our 2019 article,^3^ >35 new nanoparticle technologies have begun clinical trials. Of these new additions, 28 are lipid‐based (25 of which are for mRNA‐based vaccines). The remaining new nanoparticle technologies are indicated for cancer treatment (two pure‐drug nanoparticles are being studied in five different clinical trials), imaging applications (three trials are investigating carbon nanoparticles for imaging lymph nodes), and non‐mRNA vaccines (three protein‐based nanoparticles are being investigated as vaccines). Table 3 summarizes these new additions.

**TABLE 3:** Nanoparticle vaccines, therapies, and diagnostics which are not clinically approved and are currently active clinical trials that have appeared on the ClinicalTrial.gov database since 2019

Name (company)	Particle type	Payload	Investigated application/indication	Current ClinicalTrials.gov identifiers (phase)
*Lipid‐based*				
mRNA‐1283 (Moderna)	Lipid nanoparticle	mRNA	COVID‐19 vaccine	NCT04813796 (Ph I): Recruiting
mRNA‐1345 (Moderna)	Lipid nanoparticle	mRNA	Respiratory syncytial virus vaccine	NCT04528719 (Ph I): Recruiting
mRNA‐1647 (Moderna)	Lipid nanoparticle	mRNA	Cytomegalovirus vaccine	NCT04232280 (Ph II): Recruiting
mRNA‐1653 (Moderna)	Lipid nanoparticle	mRNA	Combined human metapneumovirus and parainfluenza virus type 3 vaccine	NCT04144348 (Ph I): Recruiting
mRNA‐2416 (Moderna)	Lipid nanoparticle	mRNA	Advanced solid tumor malignancies	NCT03323398 (Ph I/II): Recruiting
mRNA‐2752 (Moderna)	Lipid nanoparticle	mRNA	Advanced solid tumor malignancies	NCT03739931 (Ph I): Recruiting NCT02872025 (Ph I): Recruiting
mRNA‐4157 (Moderna)	Lipid nanoparticle	mRNA	Personalized cancer vaccine	NCT03313778 (Ph I): Recruiting NCT03897881 (Ph II): Recruiting
mRNA‐5671/V941 (Merck)	Lipid nanoparticle	mRNA	KRAS vaccine	NCT03948763 (Ph I): Recruiting
AZD8601 (AstraZeneca)	Lipid nanoparticle	mRNA	Personalized cancer vaccine	NCT03313778 (Ph I): Recruiting NCT03897881 (Ph II): Recruiting
MEDI1191 (MedImmune)	Lipid nanoparticle	mRNA	Advanced solid tumors	NCT03946800 (Ph I): Recruiting
DS‐5670a (Daiichi Sankyo)	Lipid nanoparticle	mRNA	COVID‐19 Vaccine	NCT04821674 (Ph I/II): Recruiting
BNT111 (BioNTech)	Size‐ and charge‐based RNA‐lipoplex nanoparticles for targeting dendritic cells	RNA that elicits immune response against four antigens	Metastatic melanoma vaccine	NCT04526899 (Ph II): Recruiting
BNT112 (BioNTech)	Size‐ and charge‐based RNA‐lipoplex nanoparticles for targeting dendritic cells	RNA that enables expression of five antigens	Prostate cancer vaccine	NCT04382898 (Ph I/II): Recruiting
BNT113 (BioNTech)	Size‐ and charge‐based RNA‐lipoplex nanoparticles for targeting dendritic cells	RNA that elicits immune response against oncoproteins E6 and E7	Head and neck cancer vaccine	NCT04534205 (Ph II): Recruiting
BNT115 (BioNTech)	mRNA‐lipoplex nanoparticles	mRNA that increases tumor associated antigen expression	Ovarian cancer	NCT04163094 (Ph I): Recruiting
BNT122/RO7198457 (BioNTech and Genentech)	Size‐ and charge‐based RNA‐lipoplex nanoparticles for targeting dendritic cells	RNA that encodes neoantigens	Colorectal cancer, melanoma, lung cancer, bladder cancer	NCT04486378 (Ph II): Recruiting NCT03815058 (Ph II): Recruiting NCT03289962 (Ph I): Recruiting
BNT141 (BioNTech)	Liver‐targeting lipid nanoparticle	mRNA that enables systemic production of IgG antibodies	Solid tumors	NCT04710043 (Ph I): Not yet recruiting
BNT151 (BioNTech)	Liver‐targeting lipid nanoparticle	mRNA that enables systemic production of IL‐2	Solid tumors	NCT04455620 (Ph I/II): Recruiting
BNT152 + BNT153 (BioNTech)	Liver‐targeting lipid nanoparticle	mRNA that enables systemic production of IL‐7 and IL‐2	Multiple solid tumors	NCT04710043 (Ph I): Not yet recruiting
CLDN6 RNA‐LPX (BioNTech)	Size‐ and charge‐based RNA‐lipoplex nanoparticles for targeting dendritic cells	RNA that encodes a receptor against CLDN6	Solid tumor	NCT04503278 (Ph I/II): Recruiting
CVnCoV (CureVac)	Lipid nanoparticle	mRNA	COVID‐19 vaccine	NCT04860258 (Ph III): Recruiting NCT04838847 (Ph III): Recruiting NCT04674189 (Ph III): Recruiting NCT04652102 (Ph II/III): Active, not recruiting NCT04515147 (Ph II): Active, not recruiting NCT04449276 (Ph I): Active, not recruiting NCT04848467 (Ph III): Not yet recruiting
CV7202 (CureVac)	Lipid nanoparticle	mRNA encoding rabies virus glycoprotein	Rabies vaccine	NCT03713086 (Ph I): Active, not recruiting
ARCT‐021/LUNAR‐COV19 (Arcturus)	Lipid‐enabled and unlocked nucleomonomer agent mRNA (LUNAR®)	mRNA	COVID‐19 vaccine	NCT04728347 (Ph II): Recruiting NCT04668339 (Ph II): Active, not recruiting NCT04480957 (Ph I/II): Recruiting
ARCT‐810/LUNAR‐OTC (Arcturus)	Lipid‐enabled and unlocked nucleomonomer agent mRNA (LUNAR®)	mRNA that enables synthesis of ornithine transcarbamylase enzyme	Ornithine transcarbamylase deficiency	NCT04442347 (Ph I): Recruiting
BP1002 (Bio‐Path Holdings)	Liposome	Antisense designed to inhibit protein synthesis of Bcl‐2	Advanced lymphoid malignancies	NCT04072458 (Ph I): Recruiting
SpFN_1B‐06‐PL + ALFQ (U.S. Army Medical Research and Development Command)	Army liposomal formulation (adjuvant)	Spike‐ferritin‐nanoparticle (vaccine)	COVID‐19 Vaccine	NCT04784767 (Ph I): Recruiting
HDT‐301 (SENAI CIMATEC)	Lipid‐Inorganic Nanoparticle (LION™); 15‐nm superparamagnetic iron oxide	repRNA	COVID‐19 Vaccine (repRNA)	NCT04844268: (Ph I): Not yet recruiting
NTLA‐2001 (Intellia Therapeutics)	Lipid nanoparticles	CRISPR/Cas9 for knockout edit to reduce transthyretin	Transthyretin amyloidosis	NCT04601051 (Ph I): Recruiting
*Pure drug*				
NanoDoce® (NanOlogy)	Large surface area microparticles (nanoparticulates)	Docetaxel	Urothelial carcinoma	NCT03636256 (Ph I/II): Active, not yet recruiting NCT04060628: Available
NanoPac® (NanOlogy)	Large surface area microparticles (nanoparticulates)	Paclitaxel	Pancreatic adenocarcinoma, lung cancer	NCT04314895 (Ph II): Recruiting NCT03077685 (Ph II): Recruiting NCT03756311: Available
*Polymeric*				
PRECIOUS‐01 (Radboud University)	Poly(lactic‐co‐glycolic acid) (PLGA) nanoparticle	Threitolceramide‐6 and the New York Esophageal Squamous Cell Carcinoma‐1 cancer‐testis antigen peptides	New York Esophageal Squamous Cell Carcinoma‐1 positive cancers	NCT04751786 (Ph I): Recruiting
*Protein‐based*				
GBP510 (SK Bioscience Co.)	Self‐assembling protein nanoparticle immunogens	Various immunogens	COVID‐19 vaccine	NCT04742738 (Ph I/II): Recruiting NCT04750343 (Ph I/II): Recruiting
NanoFlu (Novavax)	Recombinant hemagglutinin protein nanoparticle with saponin‐based Matrix‐M adjuvant	Recombinant hemagglutinin protein	Influenza vaccine	NCT04120194 (Ph III): Active, not recruiting
NVX‐CoV2373 SARS‐CoV‐2 rS/Matrix‐M1 adjuvant (Novavax)	Recombinant spike protein nanoparticle with saponin‐based Matrix‐M1 adjuvant	Recombinant spike protein	COVID‐19 vaccine	NCT04611802 (Ph III): Recruiting NCT04368988 (Ph I/II): Active, not recruiting NCT04533399 (Ph II): Recruiting NCT04583995 (Ph III): Recruiting
*Inorganic*				
SPIONS (Second Affiliated Hospital, School of Medicine, Zhejiang University)	Superparamagnetic iron oxide nanoparticles (SPIONs) with spinning magnetic field	Superparamagnetic iron oxide nanoparticles	Osteosarcoma	NCT04316091 (Ph I): Not yet recruiting
EO2002 (Emmecell)	Magnetic nanoparticles with cultured human corneal endothelial cells	Cultured human corneal endothelial cells	Corneal edema	NCT04894110 (Ph I): Recruiting
Carbon nanoparticles (YE Yingjiang)	Carbon nanoparticles	Carbon nanoparticle	Lymph node tracer in rectal cancer	NCT03550001: Not yet recruiting
Carbon nanoparticles (The First Affiliated Hospital with Nanjing Medical University)	Carbon nanoparticles	Carbon nanoparticle	Lymph node tracer in breast cancer	NCT04482803: Recruiting
Carbon nanoparticles (LI XIN‐XIANG)	Carbon nanoparticles	Carbon nanoparticle	Lymph node tracer in colorectal cancer	NCT04759820 (Ph II/III): Recruiting

*Note*: Trials are grouped by particle type.

## 5 CONCLUSION

The transformative role of lipid nanoparticles as mRNA delivery vehicles for combating COVID‐19 and their tremendous global impact during 2020 and 2021 has ushered in an unprecedented period for nanoparticle therapeutics. To date, >30 nanoparticles have been used in various clinical applications (Table 1) and >20 of these continue to be developed, with chronologically increasing activity in clinical trials (Figure 3 and Table S1). Of the 60 unapproved nanoparticle technologies currently being investigated in clinical trials, >100 active trials exist with >40 being added in this update alone (Table 2). Finally, since our previous update in 2019,^3^ there has been a massive surge in clinical introduction of new nanoparticle technologies, dominated by lipid nanoparticles for mRNA delivery; over 35 new nanoparticle technologies have entered clinical trials since 2019 (Table 3). Considering these recent updates and the global impact of nanoparticles in the clinic, especially as it relates to combating COVID‐19, the field of nanoparticle drug delivery is entering a new phase, wherein their development,^19^, ^20^ manufacturing,^21^, ^22^ and clinical utility^23^ is just beginning to scratch the surface.

## AUTHOR CONTRIBUTIONS

**Aaron Anselmo:** Conceptualization; data curation; formal analysis; supervision; writing ‐ original draft; writing‐review & editing. **Samir Mitragotri:** Conceptualization; formal analysis; supervision; writing ‐ original draft; writing‐review & editing.

## Supporting information


**Appendix S1**: Supporting information
