# Supplementary material for: Nanoparticles in the clinic: An update post COVID‐19 vaccines
Source: Bioeng Transl Med. 2021 Aug 13;6(3):e10246. doi: 10.1002/btm2.10246 (PMC8420572; doi:10.1002/btm2.10246)
Supplement: Supplementary file 1 — Appendix S1: Supporting information [file BTM2-6-e10246-s001.pdf]

Supplementary Information

Nanoparticles in the Clinic: An Update Post COVID-19 Vaccine

Aaron C. Anselmo[1*] and Samir Mitragotri[2,3*]

[1]Division of Pharmacoengineering and Molecular Pharmaceutics, Eshelman School of Pharmacy, University of North Carolina at Chapel Hill, Chapel Hill, North Carolina 27599, USA

[2]John A. Paulson School of Engineering & Applied Sciences, Harvard University, Cambridge, MA 02138, USA

[3]Wyss Institute for Biologically Inspired Engineering, Boston, MA 02115, USA

[*] Address correspondence to aanselmo@email.unc.edu or mitragotri@seas.harvard.edu

Table S1: Number of clinical trials in 2021 for each approved injectable nanoparticle. This table also includes the number of clinical trials for each approved injectable nanoparticle in 2016 and 2019 that were included in our previous 'Nanoparticles in the Clinic' reviews in 2016 [1] and 2019 [2]. These trials and nanoparticles appear/have appeared on the ClinicalTrial.gov database. Modified with permission from [1] and [2].

Name	Updates on Number of Studies on ClinicalTrials.gov Identifier
**New Additions**	
mRNA-1273<br>(Moderna)	2021:<br>mRNA-1273: 29
Tozinameran/BNT162b2 (Pfizer–BioNTech)	2021:<br>BNT162b2: 41<br>Tozinameran: 1
**Cancer**	
Doxil<br>Caelyx<br>(Janssen)	2016:<br>Doxil: 166<br>CAELYX: 90<br><br>2019:<br>Doxil: 182<br>CAELYX: 109<br><br>2021:<br>Doxil: 190<br>CAELYX: 116
DaunoXome<br>(Galen)	2016:<br>DaunoXome: 32<br><br>2019:<br>DaunoXome: 15<br><br>2021:<br>DaunoXome: 13
Myocet<br>(Teva UK)	2016:<br>Myocet: 32<br><br>2019:<br>Myocet: 35<br><br>2021:<br>Myocet: 36
Abraxane<br>(Celgene)	2016:<br>Abraxane: 295

	2019:<br>Abraxane: 432<br><br>2021:<br>Abraxane: 495
Marqibo<br>(Spectrum)	2016:<br>Marqibo: 23<br><br>2019:<br>Marqibo: 28<br><br>2021:<br>Marqibo: 30
MEPACT<br>(Millennium)	2016:<br>MEPACT: 4<br><br>2019:<br>MEPACT: 9<br><br>2021:<br>MEPACT: 3
NBTXR3<br>Hensify<br>(Nanobiotix)	2016:<br>NBTXR3: 1<br><br>2019:<br>NBTXR3: 8<br><br>2021:<br>NBTXR3: 11
Onivyde<br>MM-398<br>(Merrimack)	2016:<br>MM-398/Onivyde: 7<br><br>2019:<br>MM-398/Onivyde: 38<br><br>2021:<br>MM-398/Onivyde: 59
VYXEOS<br>CPX-351<br>(Jazz Pharmaceuticals)	2016:<br>VYXEOS: 7<br><br>2019:<br>VYXEOS: 21<br><br>2021:<br>VYXEOS: 36
**Iron-replacement**	

CosmoFer<br>INFeD<br>Ferrisat<br>(Pharmacosmos)	2016:<br>INFeD: 6<br><br>2019:<br>INFeD: 9<br><br>2019:<br>INFeD: 8
DexFerrum<br>DexIron<br>(American Regent)	2016:<br>DexFerrum: 6<br><br>2019:<br>DexFerrum: 9<br><br>2021:<br>DexFerrum: 5
Ferrlecit<br>(Sanofi)	2016:<br>Ferrlecit: 13<br><br>2019:<br>Ferrlecit: 20<br><br>2021:<br>Ferrlecit: 22
Venofer<br>(American Regent)	2016:<br>Venofer: 44<br><br>2019:<br>Venofer: 60<br><br>2021:<br>Venofer: 64
Feraheme<br>(AMAG)<br>Rienso<br>(Takeda)<br>Ferumoxytol	2016:<br>Ferumoxytol: 57<br><br>2019:<br>Ferumoxytol: 84<br><br>2021:<br>Ferumoxytol: 93
Injectafer<br>Ferinject<br>(Vifor)	2016:<br>Ferinject: 50<br>Injectafer: 8<br><br>2019:<br>Ferinject: 79

	Injectafer: 24<br><br>2021:<br>Ferinject: 93<br>Injectafer: 25
Monofer<br>(Pharmacosmos)	2016:<br>Monofer: 22<br><br>2019:<br>Monofer: 22<br><br>2021:<br>Monofer: 45
Diafer<br>(Pharmacosmos)	2016:<br>Diafer: 1<br><br>2019:<br>Diafer: 1<br><br>2021:<br>Diafer: 2
**Imaging**	
Definity<br>(Lantheus Medical Imaging)	2016:<br>Definity: 58<br><br>2019:<br>Definity: 87<br><br>2021:<br>Definity: 101
Feridex I.V.<br>(AMAG)<br>Endorem	2016:<br>Endorem: 4<br>Feridex: 2<br>No current active or recruiting studies<br><br>2019:<br>Endorem: 4<br>Feridex: 2<br>No current active or recruiting studies<br><br>2021:<br>Endorem: 4<br>Feridex: 2<br>No current active or recruiting studies
Ferumoxtran-10<br>Combidex	2016:<br>Ferumoxtran-10: 11

Sinerem<br>(AMAG)	2019:<br>Ferumoxtran-10: 24<br><br>2021:<br>Ferumoxtran-10: 29
Optison<br>(GE Healthcare)	2016:<br>Optison: 11<br><br>2019:<br>Optison: 30<br><br>2021:<br>Optison: 30
SonoVue<br>(Bracco Imaging)	2016:<br>SonoVue: 43<br><br>2019:<br>SonoVue: 72<br><br>2021:<br>SonoVue: 88
Resovist<br>(Bayer Schering Pharma)<br>Cliavist	2016:<br>Resovist: 2<br><br>2019:<br>Resovist: 2<br><br>2021:<br>Resovist: 2
**Vaccines**	
Epaxal<br>(Crucell)	2016:<br>Epaxal: 6<br><br>2019:<br>Epaxal: 6<br><br>2021: 6
Inflexal V<br>(Crucell)	2016:<br>Inflexal V: 14<br><br>2019:<br>Inflexal V: 14<br><br>2021:<br>Inflexal V: 15

**Anesthetics**	
Diprivan	2016:<br>Diprivan: 110<br><br>2016:<br>Diprivan: 162<br><br>2021:<br>Diprivan: 175
**Amyloidosis**	
ONPATTRO<br>Patisiran<br>ALN-TTR02 (Alnylam Pharmaceuticals)	2016:<br>Patisiran: 3<br><br>2019:<br>Patisiran: 11<br><br>2021:<br>Patisiran/ONPATTRO: 14
**Fungal Infections**	
AmBisome<br>(Gilead Sciences)	2016:<br>AmBisome: 50<br><br>2019:<br>AmBisome: 57<br><br>2021:<br>AmBisome: 58
**Macular Degradation**	
Visudyne<br>(Bausch and Lomb)	2016:<br>Visudyne: 52<br><br>2016:<br>Visudyne: 60<br><br>2021:<br>Visudyne: 65

References

1. Anselmo, A.C. and S. Mitragotri, *Nanoparticles in the clinic.* Bioengineering & translational medicine, 2016. **1**(1): p. 10-29.
2. Anselmo, A.C. and S. Mitragotri, *Nanoparticles in the clinic: An update.* Bioengineering & translational medicine, 2019. **4**(3): p. e10143.
